# Spirulina supplement and exercise training affect lipid droplets-related genes expression in visceral adipose tissue 

**DOI:** 10.22038/AJP.2023.22915

**Published:** 2024

**Authors:** Fariba Shahandeh, Rozita Fathi, Khadijeh Nasiri

**Affiliations:** *Department* * of Exercise Physiology, Faculty of Sport Science, University of Mazandaran, Babolsar, Iran*

**Keywords:** Exercise, Spirulina, Lipid droplets, Rab18, Gene expression

## Abstract

**Objective:**

Disruption of lipid droplets (LDs) is associated with many metabolic diseases. Spirulina, as a natural bioactive dietary supplement, along with exercise training, may improve lipid metabolism; however, their effects on LDs-regulated genes in visceral adipose tissue are still unclear. This study aimed to investigate the effects of six-week Spirulina supplementation along with exercise training on LDs regulating gene expression.

**Materials and Methods:**

Fifty-six male Wistar rats were divided into six groups: saline (control), control+Spirulina (Spirulina), aerobic interval training (AIT), AIT+ Spirulina (AIT+Spirulina), resistance training and resistance+ Spirulina. The supplement groups consumed 500 mg/kg Spirulina five days per week. The training groups performed AIT (5 times per week) and resistance training (3 times per week) for 6 weeks. LDs regulating genes expression in visceral adipose tissue (*Zw10*, *Bscl2*, *DFCP1*, *Rab18*, *Syntaxin* *18*, *Acsl3*, and *Plin2*) was analyzed by real-time PCR.

**Results:**

Spirulina and exercise training had no significant effects on the gene expression of Syntaxin18 (p=0.69) and *DFCP1 *(p=0. 84), *ACSL3* (p=0.98), or *BSCL2* (p=0.58). In addition, Spirulina was found to significantly attenuate the expression of *Plin2 *(p=0.01) and *Rab18* (p=0.01) genes compared to the control, AIT, and resistance training groups. However, *Plin2* gene expression was higher in the resistance training than the AIT. Furthermore, Spirulina decreased *ZW10* (p=0.03) gene expression in visceral adipose tissue compared to the control, AIT, and resistance training groups. Unexpectedly, Spirulina supplementation decreased the expression of these genes even more when taken without exercise training

**Conclusion:**

Spirulina supplementation and exercise training have significant effects on LDs-regulated genes in visceral adipose tissue.

## Introduction

Adipose tissue compensates for excess caloric intake by assembling and storing fatty acids in the form of triacylglycerol (TAG) in lipid droplets (LDs). LDs, which originate from the endoplasmic reticulum (ER), not only serve as a reservoir for lipid storage, but also have an important role in energy homeostasis, lipid metabolism, and cellular signaling (Sanjabi et al., 2015). 

The remodeling of LDs requires the coordination and regulation of a series of proteins, enzymes, and genes (Kumar Natarajan et al., 2017). Due to their indirect connection with vesicular transport pathways (Barbosa et al., 2015), the binding of LDs to organelles is critical for cellular homeostasis. Perilipins (PLINs) such as perilipin2 (PLIN2) and Rab proteins such as Rab18 play important roles in the biological processes of LDs, including lipolysis, lipid metabolism, tethering, and trafficking (Sztalryd and Brasaemle, 2017; Pulido et al., 2011). In addition, long-chain fatty acid CoA ligase )ACSLs(, a key enzyme of lipid metabolism, including acyl-CoA synthetase long-chain family member 3 (ACSL3), is associated with Rab18, which promotes the biogenesis of LDs (Deng et al., 2021). Deng et al. discovered that the size of LDs and the content of TAG are regulated by the PLIN2/Rab18/ACSL3 protein complex (Deng et al., 2021). 

It is well documented that disruption of lipid storage and LDs release leads to many metabolic diseases such as diabetes, lipodystrophies, and insulin resistance (Krahmer et al., 2013). Spirulina, a natural product enriched with bioactive substances, has gained a growing interest in the prevention and treatment of metabolic diseases in the last decade (Grosshagauer et al., 2020). In this context, Spirulina has been shown to improve serum lipids, glucose, appetite, and body weight (Zeinalian et al., 2017; Huang et al., 2018; Hernández-Lepe et al., 2019b).

On the other hand, lifestyle changes improve the physiology and morphology of adipose tissue, which eventually lead to improvements in metabolic diseases including insulin resistance (Kolahdouzi et al., 2019). The underlying mechanism is not fully understood, but there is evidence that exercise training improves adipose tissue metabolism by increasing angiogenesis and decreasing adipose tissue inflammation and adipocyte size and number (Kolahdouzi et al., 2019) which may be related to the metabolism of LDs (Li et al., 2021). In skeletal muscle, exercise training has been shown to reduce the diameter and density of the subsarcolemmal LD population, but not the intramyofibrillar regions (Li et al., 2014). Shepherd et al. showed that exercise training in obese men increased PLIN2 in skeletal muscles and LD content in contact with mitochondria (Shepherd et al., 2017). However, these effects in adipose tissue LDs have not yet been investigated. 

The synergistic effects of natural supplements and exercise training could improve physical health and weight management and have attracted increasing interest worldwide. It has been documented that Spirulina intake in combination with exercise training improves body composition, blood lipid profiles, and cardiorespiratory fitness (Hernández-Lepe et al., 2019a). However, as aforementioned, the effects of exercise training on LDs content and related proteins and genes in adipose tissue are elusive. Therefore, this study investigated the effects of different exercise training modalities in synergy with Spirulina ingestion on LDs metabolism-related genes in visceral adipose tissue. 

## Materials and Methods

**Animal care**

For this study, 4-week-old Wistar rats which were purchased from Pasteur Institute of Iran, were used. Based on the guidelines and use of laboratory animals (Council, 2010), animal care and ethical principles were approved by the Ethics Committee of the University of Mazandaran (IR.UMZ.REC.1401.046). Accordingly, 56 male Wistar rats were kept in a standard cage (4 rats per cage) at a room temperature of 22±1^°^C, a humidity of 55%, and a light-dark cycle of 12 to 12 hr. After one week of adaptation to the environment, animals were divided into six groups based on their body weight: 

1) control with saline (control), 

2) control with Spirulina (Spirulina), 

3) aerobic interval training plus saline (AIT);

4) AIT plus spirulina (AIT+Spirulina),

5) resistance training plus saline (resistance) 

and 6) resistance plus Spirulina (resistance+ Spirulina). 

Body weight was measured weekly. After six weeks of training, rats were anesthetized and sacrificed. The visceral adipose tissue was then removed and stored at -80^∘^C.

**Exercise training protocols**

**AIT**

Animals in AIT groups, after a 1-week familiarization period on a treadmill (10 min, 5 times a week), participated in a 6-week training period (5 times a week). AIT protocol consisting of 10 min warm-up (50-60% maximal oxygen uptake (VO2max)), 4×7-min bouts at a speed of 25 m/min with 15% incline (equivalent to 85-90% VO2max), interspersed with 3×3-min active recovery at a speed of 15 m/min (equivalent to 50-60% VO2max), and 10-min cool-down (Qin et al., 2020). The duration, speed, and incline were gradually increased over the course of 5 weeks. The speed started at 15 m/min and was gradually increased by 1-2 m/min per session, reaching 25 m/min at the end of week 5. AIT was performed on a motorized treadmill with minimal use of electric shocks (Cai et al., 2019). The exercise training protocols, AIT and resistance training, were performed during the dark phase of the rats’ lives (active phase). Similar to exercise training, animals in the control groups were kept outside the cage to reduce stress.

**Resistance training**

Animals in resistance training groups were subjected to 6 weeks of progressive resistance training using a one-meter ladder with 26 rungs at an angle of 80° (3 times a week). The rats climbed the ladder with a weight attached to their tails. The overall resistance training program consisted of 3 phases: in phase 1; familiarization, the animals climbed the ladder without the weight attached to their tail for 5 weeks (4-5 repeated trials interspersed with a 2-min rest between trials). In phase 2, the overload phase, the animals climbed the ladder in 3 sessions, with weights up to 30% of body weight in the first session and up to 50% of body weight in the second and third. Each training session consisted of 2 sets of 5 repetitions, interspersed with 1-3 min of recovery between repetitions and sets, respectively. 

In phase 3, from the fourth to the last training session, the animals performed resistance training with a load of 50% of their body weight, which was gradually increased to 200% of their body weight. Each training session consisted of 3 sets of 6 repetitions, interspersed with 1-and 3-min recovery between repetitions and sets, respectively. Warm-up and cool-down (climbing the ladder twice without load) were performed before and after each training session (Talebi-Garakani and Safarzade, 2013).

**Spirulina supplement**

Spirulina powder was obtained from Exir Gole-Sorkh Pharmaceutical Company, Iran. Considering the body weights of rats, 500 mg/kg of Spirulina mixed with saline was administered to the animals by gavage. The supplementation groups received Spirulina 5 days per week for 6 weeks. In the supplementation plus training groups, Spirulina supplementation was administered 30 min before exercise training sessions (Brito et al., 2020).

**Real-time quantitative PCR**

Total RNA was isolated from epididymal adipose tissue using an RNA extraction kit (Denazist, Iran). Then, RNA samples were treated with the DNaseI enzyme (cinaclone, IRAN). The quantity and quality of extracted RNA were assessed by electrophoresis and UV spectrophotometry (Nano Mabna Iranian, Iran). The cDNA synthesis was performed using the Yakta Tajhiz Azma kit (Tehran, Iran). The relative amount in real-time PCR was analyzed by increased fluorescence, resulting in SYBR Green color binding. During this phase, the polymerase chain reaction for the genes of *Plin2*, *Syntaxin18*, *ACSL3*, *Rab18*, *Double* *FYVE-containing protein 1 *(*DFCP1*), *Berardinelli-Seip congenital lipodystrophy 2 *(*Bscl2*), *ZW10* and the reference gene *β*-*actin* was performed using SYBR Green PCR Master Mix (Ampliqon, Denmark) in the Corbett Rotor-Gene 6000. The thermal cycle of real time PCR amplifications was 95^∘^C for 15 min, followed by 45 cycles at 95^∘^C for 30 sec and 57^∘^C for 30 sec, and 30 sec at 72^∘^C.The expression of mRNA for each target gene was normalized to that of *β*-*actin* . Melting curves were carried out after the amplification to avoid nonspecific products. The target gene expression was calculated using the livak method (*2*^-ΔΔCT^). The primers used in our study are summarized in Table 1.

**Table 1:** Characterization of specific primers of the adipose tissue genes

**Genes**	**Forward primer** **(5'-…-3')**	**Reverse primer** **(5'-…-3')**	**Accession number**	**Production length** **(bp) **
***Zw10***	GCTGCCATTCACCATAACAACT	AGTGGTCGTGCCATCACAAAG	NM_001024801.1	108
***Bscl2***	TTAGAGCTTGAGCTGCCAGAAT	GAGCGTGAAGAAGTGGAGATGA	NM_001012171.2	110
***Double FYVE-containing protein 1(DFCP1)***	TACAAGGGAACGAGGACATACAA	ATCTGGTCATCAGGGATCTCAC	NM_001106743.1	173
***Rab18***	ACTCTGAAGATCCTCATCATTGG	AAGTTTAGCCTTATTTCCATCCAC	NM_001012468.1	156
***Syntaxin 18***	CACAGAGCGAGACCAGATAGAC	GGAATGAATCTCCTTGTGGGCT	NM_001012151.1	103
***Acsl3***	GTAACAGCAGTGAAATGGAAAACG	GGGTCAGGGCTCAAACGAAT	NM_057107.2	112
***Plin2***	CGGCGTTCCGCAATGTTA	AGCCAGTTGAGAGGCGTGTT	NM_001007144.3	139
***β-Actin***	GTGTGACGTTGACATCCGTAAAGAC	TGCTAGGAGCCAGGGCAGTAAT	NM_031144.3	119

**Figure 1:**
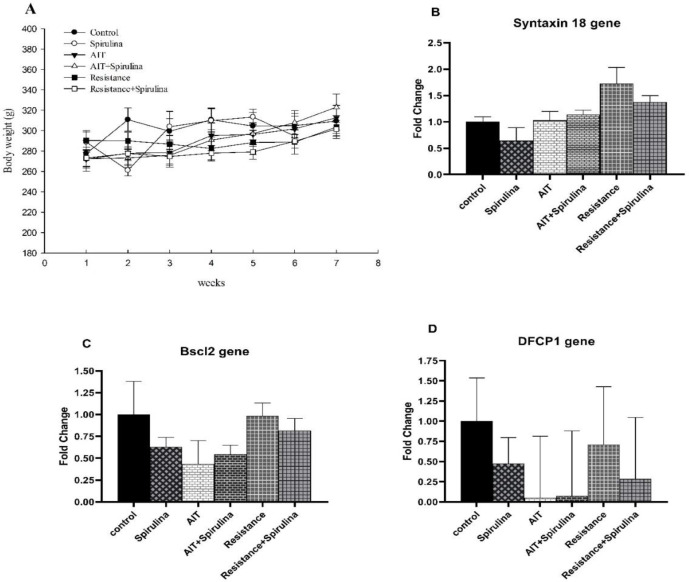
Effects of Spirulina supplement and exercise training on body weight and lipid droplets (LDs) genes expression (*Syntaxin18*, *BSCL3* and *DFCP1*) in visceral adipose tissue. To establish a comparison between groups, 2 ways ANOVA with the LSD post hoc test at significant level of 0.05 was used. AIT; aerobic interval training. Data are represented as mean±SEM.

**Figure 2:**
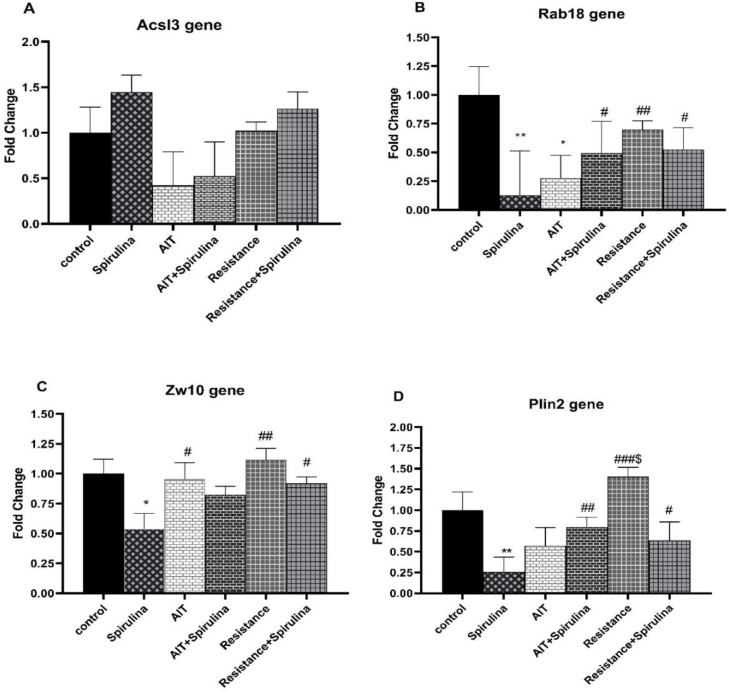
Effects of Spirulina supplement and exercise training on Lipid droplets genes expression (*ACSL3*, *Rab18*, *ZW10* and *plin2*) in visceral adipose tissue. To establish a comparison between groups, 2-way- ANOVA with the LSD *post hoc* test at significant level of 0.05 was used. AIT; aerobic interval training. ^* ^and^ **^vs. control; ^#, ## ^and^ ###^vs. Spirulina; ^$^vs. AIT. Data are presented as mean±SEM.

**Statistical analysis**

Data were compared in a two-ways ANOVA (Spirulina×exercise). To establish a comparison between groups, the LSD *post hoc* test was used. Data are expressed as the mean±standard error. Significant differences were indicated at p<0.05.

## Results

The results showed no significant differences in body weight between groups at baseline and after each week of the planned protocol (p>0.05, Figure 1A).

The findings indicated that there were no significant effects of Spirulina (F(1,21)=0.53, p=0.47), exercise (F(2,21)=2.07, p=0.15), or the interaction of spirulina and exercise (F(2,21)=0.36, p=0.69) on the gene expression of visceral adipose tissue *Syntaxin 18* (Figure 1B). The results also revealed that the *BSCL2* gene in adipose tissue showed no response to Spirulina or exercise training after 6 weeks of the designed protocols (Figure 1C).

Compared to the control group, the results showed that *DFCP1* gene expression in visceral adipose tissue decreased in response to Spirulina and exercise training. However, it did not reach a statistically significant level (supplement: (F(1,19)=0.19, p=0.66), exercise: (F(2,19)=2.31, p=0.12), and interaction: (F(2, 19)=0.17, p=0. 84) (Figure1D). Quantification results showed that AIT and Spirulina plus AIT non-significantly decreased *ACSL3* gene expression in adipose tissue by 0.5-fold, but Spirulina increased *ACSL3* gene expression by 0.5-fold. Overall, no significant effects of Spirulina supplementation (F(1,20)=0.50, p=0.48), exercise (F(2,20)=2.88, p=0.07), or interaction (F(2,20)=0.017, p=0.98) on *ACSL3* gene expression were detected (Figure 2A). 

*Rab18* gene expression in visceral adipose tissue was significantly affected by the interaction of Spirulina and exercise (F(2,22)=5.54, p=0.01), but Spirulina and exercise individually had no effect on *Rab18* gene expression (F(1,22)=3.18, p=0.08), and F(2, 22)=1.04, p=0.37), respectively. Further *post-hoc* analysis showed that Spirulina decreased *Rab18* gene expression by 85% compared to the control group (p=0.001). Spirulina also significantly decreased *Rab18* gene expression compared to AIT plus Spirulina (85% vs. 50%, p=0.022), resistance training (85% vs. 65%, p=0.008), and resistance training plus spirulina (85% vs. 50%, p=0.017). AIT significantly decreased *Rab18* gene expression by 75 % compared to the control group (p=0.039, Figure 2B). 

As for *ZW10* gene expression, Spirulina supplementation significantly decreased *ZW10* gene expression (F(1,21)=5.33, p=0.03). However, no significant effects of exercise (F(2,21)=1.70, p=0.20) or the interaction of exercise and supplementation (F(2,21)=1.10, p=0.35) were detected. *Post-hoc* analysis showed that spirulina decreased *ZW10* gene expression by approximately 50% in comparison with the control group (p=0.025). The gene expression of *ZW10* was significantly lower in the Spirulina supplement group than the AIT (p=0.018), resistance (p=0.005) and resistance training+ spirulina groups (p=0.026) (Figure 2C). 

As shown in Figure 2C, Spirulina supplementation had a significant effect on *Plin2* gene expression in visceral adipose tissue (F(1, 22)=6.55, p=0.01). However, the effects of exercise training did not reach a significant level (F(2,22)= 2.23, p=0.13). Furthermore, the synergistic effects of Spirulina supplementation and exercise training were significant (F(2,22)=4.35, p=0.02). To examine the differences between the groups, further analysis showed that Spirulina supplementation significantly reduced *Plin2* gene expression by 80% compared to the control group (p=0.006). 

The data also revealed that spirulina consumption alone had a stronger effect on *Plin2* gene expression than exercise training with and without Spirulina consumption (p<0.05). In addition, resistance training increased *Plin2* gene expression by approximately 40% compared to the control group, which was not significant but was significantly greater than that of AIT (p=0.031) (Figure 2D). 

## Discussion

The main findings of the present study indicate that Spirulina supplementation stimulates LDs-regulated genes in visceral adipose tissue. However, the Spirulina-induced changes in LDs-related genes were attenuated by the synergistic effects of exercise training and Spirulina supplementation. Furthermore, as we have shown, it appears that the beneficial effects of exercise training on adipose tissue metabolism are related to the regulation of LDs-related gene expression. 

*PLINs* are the family members of cytoplasmic LDs genes that regulate neutral lipid storage and hydrolysis (Brasaemle, 2007). *Plin2* is predominantly expressed by adipocytes and is located in the cytoplasmic LDs (Brasaemle et al., 1997). It has been shown that the *Plin2* gene may prevent high-fat diet-induced obesity by decreasing food intake and adipocyte size (McManaman et al., 2013). Our data showed that Spirulina decreased *Plin2* gene expression by 80% compared to the baseline. When Spirulina, AIT, and resistance training were used together, *Plin2* gene expression decreased to ~50-70%. However, separately, resistance training increased, but AIT decreased the gene expression of *Plin2* in visceral adipose tissue. In Plin2 knockdown mice, it has been observed that the mRNA level of Peroxisome proliferator-activated receptor-γ coactivator 1α was non-significantly higher in epididymal fat pads, while Uncoupling Protein 1 (UCP1) was significantly increased (McManaman et al., 2013). It is of note that crown-like structures, which are histologic hallmarks of inflammation and are enriched in LDs and coated with Plin2, significantly decreased in the absence of *Plin2* (McManaman et al., 2013). Although the underlying mechanism of the effects of Spirulina and exercise training on the changes of *Plin2* gene expression in visceral adipose tissue has not been determined, it seems that Spirulina (Fujimoto et al., 2012) and AIT (Kolahdouzi et al., 2019) prevent macrophage infiltration into the visceral fat, which is associated with decreased crown-like structures. It has been reported that sprint interval training and moderate continuous training increased *Plin2* in type I muscle fibers without any significant effects on LD content; however, the portion of LD in contact with mitochondria increased (Shepherd et al., 2017). In our study, we did not analyze the protein expression of Plin2 in adipose tissue to show the precise effects of exercise training or Spirulina on LDs regulating proteins; therefore, future investigations are required here. Nonetheless, in the skeletal muscles, it has been shown that the Plin2 protein in trained subjects is higher than untrained subjects (Koves et al., 2013). 

Plin2 together with Rab18 regulate LDs size and number (Deng et al., 2021). Rab18 affects TAG accumulation and LDs dynamics, and its overexpression increases the number of LDs (Deng et al., 2021). We reported here that the synergistic effect of Spirulina and exercise training on *Rab 18* gene expression was significant (Figure 2B). In fact, Spirulina decreased *Rab18* gene expression by about 85% compared to the control groups. But this reduction in combination with AIT and resistance training was lower (50% vs. 65%, respectively). In addition, AIT decreased *Rab18* gene expression by about 70% in comparison with the control groups. It can be speculated that AIT and Spirulina, independently, are more effective than the synergic effects of both on *Rab18* gene expression. It seems that Spirulina and exercise training, by reducing *Plin2*, attenuate *Rab18*. However, the mechanism behind this reduction has not been identified. Previously, it was found that *Plin2* depletion reduced *Rab18* in LDs (Deng et al., 2021). Also, insulin-induced Rab18 stimulates fat accumulation in adipocytes via phosphatidylinositol 3-kinase (Pulido et al., 2011). Exercise training reduces insulin levels (Kolahdouzi et al., 2019) which may suppress Rab18 and eventually increase the size of LDs in adipose tissue.

More recently, Deng et al. suggested that the Plin2/Rab18/ACSL3 complex regulates the homeostasis of LDs, with Plin2 promoting ACSL3 to the LD surface for acyl-CoA synthesis by facilitating and stabilizing Rab18-targeted LDs (Deng et al., 2021). Our data showed that the effects of exercise training and Spirulina on *ACSL3* gene expression were not significant, but AIT and AIT plus spirulina reduced *ACSL3* gene expression. Overexpression of *Rab18* is associated with *ACSL3* in LDs (Deng et al., 2021). It has been suggested that Rab 18 affects TAG accumulation by regulating ACSL3 in LDs (Deng et al., 2021). It has also been reported that overexpression of BSCL2/Seipin reduces lipid storage to some extent by enhancing lipolysis (Cui et al., 2012). Accordingly, we reported here that Spirulina and exercise training decreased *BSCL2* gene expression (Figure 1C). BSCL2 facilitates the transport of ACSL3 protein from the ER into expanding LDs (Wang et al., 2016). Increasing adipose tissue oxygenation by stimulating adipose tissue capillary density alters adipose tissue inflammation (Elias et al., 2012; Song et al., 2016). Consistent with this assumption, Kolahdouzi et al. reported that AIT decreased inflammatory macrophages and increased anti-inflammatory macrophages, which were associated with increased capillary density and decreased adipocyte size and density (Kolahdouzi et al., 2019). 

*Rab18* interacts with tethering factors such as *ZW10* and *Syntaxin 18*, which in return enhances the binding between ER and LD for the development of LDs growth (Xu et al., 2018). We have also observed that Spirulina decreased the gene expression of *Syntaxin 18* in visceral adipose tissue, while exercise training increased the gene expression of *Syntaxin 18*, although this was not statistically significant. However, Spirulina significantly decreased *ZW10* gene expression compared to the control and exercise training groups. Due to the lack of experimental evidence, the mechanisms by which exercise training or natural supplementation regulates LDs-related genes have not yet been investigated. It is evident that the GTP-linked form of Rab18 directly links to ZW10 and regulates the growth and storage of LDs (Xu et al., 2018). Thus, it can be concluded that Spirulina affects adipose tissue cell size in part by regulating genes related to the association of LDs with ER. ER and LD connections are enhanced by overexpression of DFCP1. DFCP1, as an effector of Rab18, intervenes in the association of ER and LDs with the complex Rab18-ZW10 (Li et al., 2019). Overexpression of DFCP1 increases the size of LDs, and depletion of DFCP1 decreases the size of LDs (Li et al., 2019). An increase in ER and LD compounds induced by DFCP1 or Rab18 overexpression depends on Rab18 and DFCP1, respectively (Zhao et al., 2017). In our study, gene expression of *DFCP1* non-significantly decreased in response to exercise training and Spirulina administration, which is consistent with the reduction of *Rab18*, which may explain the close relationship between *Rab18* and *DFCP1*. It has also been shown that BSCL2 depletion reduces the binding between ER and LD induced by Rab 18 or DFCP1 overexpression (Li et al., 2019). The explanation for this finding is that the density of the crown-like structure has a positive correlation with adipocyte size (Weisberg et al., 2003), and it has been reported that Plins in adipose tissue increased in large adipocytes (Laurencikiene et al., 2011). In contrast, exercise training reduces crown-like structure and adipocyte size (Kolahdouzi et al., 2019) as well as lipogenic markers in visceral adipose tissue, which are associated with an improvement in the mitochondrial function of visceral adipose tissue (Rocha‐Rodrigues et al., 2017). Another possible explanation relates to the exercise training-induced activation of AMP kinase (AMPK) in white adipose tissue (Higa et al., 2014), resulting in decreased lipogenesis enzyme activity (Higa et al., 2014) and improved fat oxidation (Ceddia, 2013). AMPK activity is the major regulator of protein levels in response to exercise training adaptation in visceral adipose tissue (Takekoshi et al., 2006). In this regard, it was observed that adipose tissue mass increased in AMPK knockout mice, likely due to increased lipogenesis (Villena et al., 2004).

Even though it is not clear how Spirulina might affect the metabolism of adipose tissue, a lot has been learned about how exercise training affects the metabolism of adipose tissue. However, since one of the limitations of the present study is not the evaluation of protein levels , future studies are needed. The strength of this study is that our data revealed another part of the effects of exercise training on adipose tissue at the cellular level, namely gene expression related to LDs. Nevertheless, our study has some limitations. First, we did not analyze protein levels or adipocyte size, which could explain the exact mechanism of exercise training on adipose tissue remodeling. Second, because the aforementioned genes change with obesity and overeating, we were not able to induce obesity before the intervention. We believe that exercise training or Spirulina supplementation may promote the spread of LDs. Thus, future studies should consider these limitations in their planned interventions.

In conclusion, our research shown that Spirulina and exercise alter the expression of genes that regulate the metabolism of LDs. On the level of gene expression, however, the synergistic benefits of exercise training and spirulina appear doubtful in light of our findings. Alone, exercise training and Spirulina appear to be more effective at regulating LDs-related genes than their combination. Further research is required to confirm these findings.

## Conflicts of interest

The authors have declared that there is no conflict of interest.
